# Intravenous delivery of adeno-associated virus 9-encoded IGF-1Ea propeptide improves post-infarct cardiac remodelling

**DOI:** 10.1038/npjregenmed.2016.1

**Published:** 2016-06-09

**Authors:** Enrique Gallego-Colon, Maria Villalba, Joanne Tonkin, Francisco Cruz, Juan Antonio Bernal, Luis J Jimenez-Borregureo, Michael D Schneider, Enrique Lara-Pezzi, Nadia Rosenthal

**Affiliations:** 1 Imperial Centre for Translational Research, National Heart and Lung Institute, Faculty of Medicine, Imperial College London, London, UK; 2 Cardiovascular Development and Repair Department, Centro Nacional de Investigaciones Cardiovasculares (CNIC), Madrid, Spain; 3 Australian Regenerative Medicine Institute/EMBL Australia, Monash University, Melbourne, VIC, Australia

## Abstract

The insulin-like growth factor Ea propeptide (IGF-1Ea) is a powerful enhancer of cardiac muscle growth and regeneration, also blocking age-related atrophy and beneficial in multiple skeletal muscle diseases. The therapeutic potential of IGF-1Ea compared with mature IGF-1 derives from its local action in the area of synthesis. We have developed an adeno-associated virus (AAV) vector for IGF-1Ea delivery to the heart to treat mice after myocardial infarction and examine the reparative effects of local IGF-1Ea production on left ventricular remodelling. A cardiotropic AAV9 vector carrying a cardiomyocyte-specific IGF-1Ea-luciferase bi-cistronic gene expression cassette (AAV9.IGF-1Ea) was administered intravenously to infarcted mice, 5 h after ischemia followed by reperfusion (I/R), as a model of myocardial infarction. Virally encoded IGF-1Ea in the heart improved global left ventricular function and remodelling, as measured by wall motion and thickness, 28 days after delivery, with higher viral titers yielding better improvement. The present study demonstrates that single intravenous AAV9-mediated IGF-1Ea Gene Therapy represents a tissue-targeted therapeutic approach to prevent the adverse remodelling after myocardial infarct.

## Introduction

Cardiovascular diseases (CVD) are mainly disorders of the blood vessels and the heart, and are the number one cause of death globally.^1^ Among the most common types of CVD, ischemic injury leading to left ventricular (LV) dysfunction is a major cause of mortality.^2,3^ Current interventions^4^ for ischemic injury are targeted at physical removal of the obstructions of the heart vessels to restore blood flow to ischemic tissue. Paradoxically, reperfusion of the tissue leads to increased radical oxygen species production, which potentially causes adverse effects on the surviving myocardium.^5^ Over the past decades, treatment for myocardial ischemia has made significant progress; unfortunately, myocardial infarction remains an unsolved therapeutic target.

Insulin-like growth factor-1 (IGF-1),^6^ an FDA-approved treatment for short-stature in children, has been extensively tested for its therapeutic properties in the resolution of tissue injury.^7^ In adults, serum IGF-1 is mainly secreted by the liver and functions as an endocrine mediator of growth and metabolism.^8^ IGF-1 is also transiently produced upon injury on most cell types to act locally in an autocrine and paracrine manner where it regulates cellular survival, proliferation and differentiation, but the molecular basis of these diverse functions is not well understood.^9^ Using mouse transgenesis supplemental expression of a locally acting IGF-1 Ea propeptide (IGF-1Ea) promoted efficient tissue repair of skeletal muscle without scar tissue formation by increasing fibre size and muscle size hypertrophy, reducing cachexia, ageing, increasing the number of myogenic progenitors and promoting the fusion of nascent myocytes.^10–12^ Furthermore, it has been demonstrated that IGF-1 accelerates muscle regeneration by modulating inflammatory cytokines and macrophage polarisation during muscle regeneration.^13,14^ In the heart, transgenic IGF-1Ea restored heart functionality post-infarct, increasing anti-apoptotic signalling and reducing scar formation facilitated by the modulation of the innate immune cell populations soon after infarction, favouring a reduction in inflammatory myeloid cells, modulating cytokine expression and matrix turnover within the first 7 days after infarct.^15,16^ These studies suggest how supplemental IGF-1Ea, expressed locally, can modulate the stimulus of injury or disease, by mobilising immune cells to target and rebuild damaged tissues in a new paradigm of self-renewal. However, the heart-specific transgene is expressed from the time of cardiac myogenesis onward, and does not simulate delivery to humans in the clinic.

In the present study, we tested adeno-associated virus (AAV)-9 as a therapeutically relevant vector for promoting heart repair with IGF-1Ea. In recent years, the use of AAV has proved to be the most promising gene delivery system for the transfer of genes to post-mitotic cells *in vitro* and *in vivo*.^17–19^ So far, high efficiency whole-heart gene delivery in small animals has only been effectively achieved with AAV serotype 9.^20,21^ We therefore exploited intravenous delivery of an AAV9-based IGF-1Ea gene vector to ameliorate the adverse effects of the heart remodelling after ischemic injury, as a clinically applicable delivery system.

## Results

### Characteristics of intravenous AAV9.IGF-1Ea gene transfer

**Figure 1:**
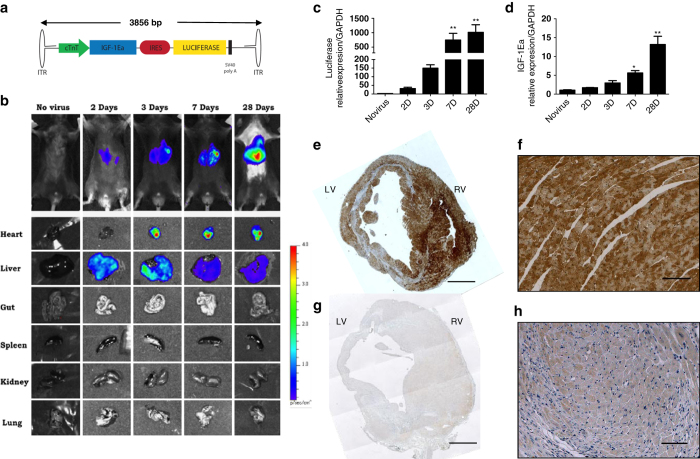
Time course of AAV9 vector mediated IGF-1Ea gene expression in the murine heart after intravenous injection. (**a**) Schematic representation of recombinant AAV vector. The construct contains the mouse IGF-1Ea gene (462 bp) and an internal ribosomal entry site (IRES) followed by the firefly luciferase reporter gene. The expression of this bi-cistronic DNA is under the control of the cardiac troponin promoter (cTNT), and the SV40 polyA signal, flanked by inverted terminal repeats (ITR), shown as hairpin loops. The AAV vectors (AAV9.GFP and AAV9.IGF-1Ea) were packaged into AAV9 capsids. (**b**) Bioluminecesce imaging of uninjured mice illustrating the distribution of luciferase after AAV9.IGF-1Ea administration (3.5×10^11^ genome copies). *Ex vivo* bioluminescence images at indicated times of various tissues (heart, liver, gut, spleen, kidney and lungs) after IGF-1Ea cardiac gene transfer. Quantitative qPCR analysis of (**c**) luciferase and (**d**) IGF-1Ea in the heart at several timepoints after injection of I/R+IGF1Ea.10^11^. *n*=3. *P<0.05, **P<0.001. One-way ANOVA with Dunnett’s Multiple Comparison test. I/R group (no viral treatment group) as control group. (**e**–**h**) Immunohistochemistry of luciferase expression in heart sections 28 days after I/R in **e**, **f** AAV9.IGF-1Ea 3.5×10^11^ GC injected 5 h after I/R. (**e**) whole heart and (**f**) 40×magnification. (**g**, **h**) no virus (I/R) group, (**g**) whole heart and (**e**) ×40 magnification. (**d**, **e**) Scale bar, 1 mm. (**e**–**g**) Scale bar, 250 μm. LV, left ventricle; RV, right ventricle. Mean values±s.e.m. are shown.

To evaluate the therapeutic potential of AAV9-mediated IGF-1Ea gene transfer, we constructed an AAV9 vector encoding the IGF-1Ea mouse complementary DNA (cDNA), the expression of which was driven by the cardiac troponin promoter (cTNT). The plasmid also contained an internal ribosomal entry site for the luciferase gene (Figure 1a), allowing gene delivery to be readily assayed. We first assessed transduction levels by administering 3.5×10^11^ GC of AAV9.IGF-1Ea in a single intra-femoral vein injection to C57BL/6 mice. The *in vivo* expression of luciferase was evaluated at different time points. Significant expression of luciferase *in vivo* was seen in the heart at 7 days post-injection. Ectopic expression in the liver was detected albeit at much lower levels than in the heart, as early as 2 days post-injection (Figure 1b), temporarily overriding the specificity of the cTNT promoter. *Ex vivo* imaging detected luciferase expression in the heart as early as 3 days post-injection (Figure 1c and Supplementary Figure S1A,B). Further analysis revealed that the emission of photons at 3 days may be blocked by the tissue thickness and composition of the chest (Supplementary Figure S1C). Quantitative analysis of luciferase and IGF-1Ea expression verified increased levels of messenger RNA (mRNA) by 3 days post-injection, however, IGF-1Ea levels were not significantly induced until 7 days post-injection (Figure 1c,d). By contrast, there was no difference in IGF-1Ea expression between control and AAV9.IGF-1Ea groups in the liver and lung (Supplementary Figure S2A,B). Immunohistochemistry for luciferase confirmed extensive expression throughout the heart with a mosaic pattern (Figure 1e–h) typical of AAV gene transfer.^22^ In conclusion, cardiac expression of IGF-1Ea starting between 3 and 7 days after injection exhibited robust and sustained gene expression, and bioluminescent imaging of luciferase was a faithful indicator of co-expressed IGF-1Ea.

### AAV9.IGF-1Ea Gene transfer improves global left ventricular function after ischemia/reperfusion

**Figure 2:**
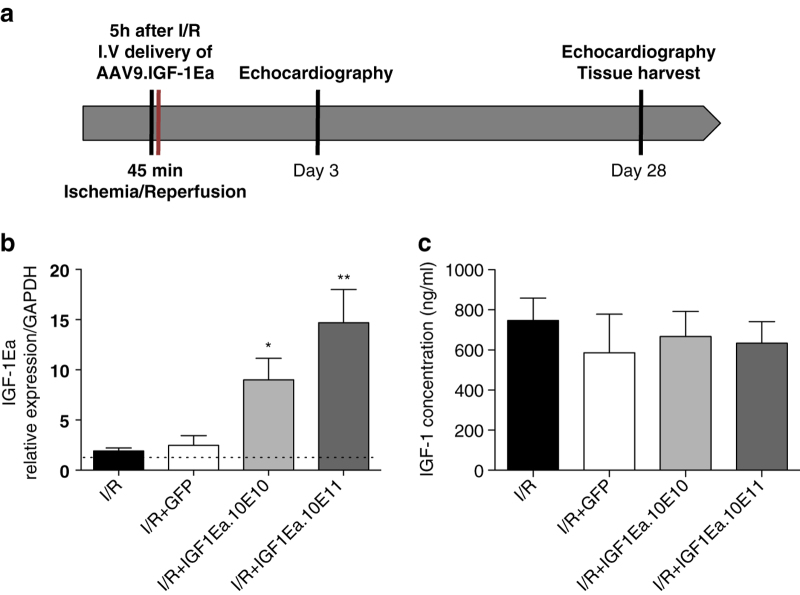
AAV9.IGF-1Ea gene transfer experimental design. (**a**) AAV9.IGF-1Ea was administered intravenously, 5 h after Ischemia/Reperfusion (I/R) and echocardiographic measurements were performed at 3 days and 28 days after I/R. Organs were collected at 28 days for analysis. (**b**) IGF-1Ea mRNA levels in the heart 28 days after cardiac gene transfer were quantified by qRT-PCR. *n*=5–12 per group. (**c**) IGF-1 Serum levels 28 days post-I/R. *n*=6–12 per group. *P<0.05, **P<0.001. One-way ANOVA with Dunnett’s Multiple Comparison test. I/R group (no viral treatment group) as control group. Mean values±s.e.m. are shown.

To test the efficacy of virally delivered IGF-1Ea in ameliorating the response to cardiac injury, mice underwent myocardial ischemia for 45 min followed by reperfusion. Four groups of animals were compared; mice with no viral treatment (ischemia/reperfusion (I/R)) and a group receiving 3.5×10^11^ GC AAV9.GFP (I/R+GFP) were used as negative-control groups; two different viral titres groups for AAV9.IGF-1Ea were tested, 3.5×10^10^ GC and 3.5×10^11^ GC (I/R+IGF-1Ea.10^10^ and I/R+IGF-1Ea.10^11^), respectively. AAV9 administration was performed 5 h after surgery by intrafemoral vein injection and mice were killed 28 days after injection (Figure 2a). A significant dose-dependent increase in IGF-1Ea mRNA levels was observed in those groups that received the AAV9.IGF-1Ea treatment (Figure 2b). ELISA quantification revealed no differences in IGF-1 serum levels between groups (Figure 2c), confirming, our previous study, that IGF-1Ea strongly adheres into extracellular matrix, retaining the propeptides locally, preventing their release into the circulation, and minimising the potential hazard of ectopic effects.^23^

**Figure 3:**
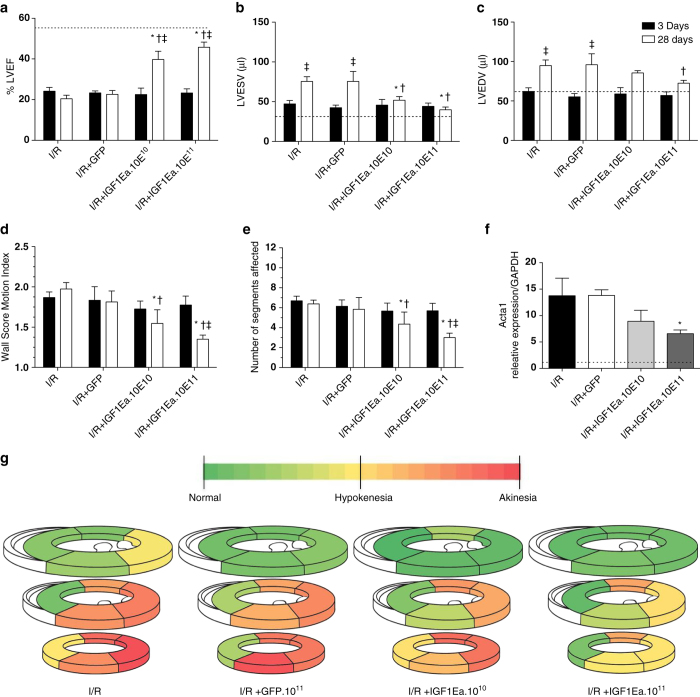
IGF-1Ea gene transfer improves cardiac function and reduces LV dilation after I/R. (**a**–**c**) Animals were analyzed by echocardiography 3 and 28 days post-I/R. (**a**) LV ejection fraction and (**b**, **c**) LV end-systolic and end-diastolic volumes, (**d**) wall score motion index (WSMI) and (**e**) number of segments affected were determined. (**f**) mRNA levels of α-skeletal actin in the remote myocardium analyzed by qRT-PCR. (**g**) Segment analysis based on wall motion and thickening. Segment scores are colored coded from green to red representing: normal or normal=green, hypokinesis=yellow, akinesis=red. Black bars: 3 days after I/R, white bars: 28 days port I/R. n=7-13. *P<0.05 I/R versus AAV9.GFP, AAV9.IGF-1Ea groups. One-way ANOVA with Dunnett's Multiple Comparison post-test. ^†^*P*<0.05. Two-way ANOVA to compare the 4 groups with Bonferroni post-tests. ^‡^*P*<0.05. Two-way ANOVA to compare 3 days versus 28 days with Bonferroni post-tests. The dashed line shows the ejection fraction of basal group. LVEF, left ventricular ejection fraction; LVEDV, left ventricular end-diastolic volume; LVESV, left ventricular end-systolic volume. Mean values ± s.e.m. are shown.

Echocardiographic analysis 28 days after I/R revealed improved left ventricular ejection fraction in mice treated with AAV9.IGF-1Ea (I/R+IGF1Ea.10^10^ and I/R+IGF-1Ea.10^11^) compared with untreated mice and mice treated with AAV9.GFP (Figure 3a and Supplementary Table S1). Left ventricular (LV) volume, as measure of LV dilation, in IGF-1Ea overexpressing mice at 28 days was also improved compared with LV volume at 3 days in the same group. In addition, AAV9.IGF-1Ea treated mice showed reduced LV dilation 28 days post-I/R (Figure 3b,c and Supplementary Table S1) compared with I/R and I/R+GFP groups. All four groups showed similar functional worsening in cardiac parameters and chamber dilation 3 days post-I/R regardless of the treatment, suggesting that initial infarct size was analogous among all groups (Supplementary Table S1). Notably, functional improvement was more evident in those groups where the highest AAV9.IGF-1Ea viral titre was administered.

### Regional left ventricular wall motion is enhanced by 28 days after AAV9.IGF-1Ea gene transfer

We performed quantitative evaluation of LV regional wall motion after infarction using echocardiography. Consistent with the LV functional data, an improvement in the wall score motion index was observed in those groups that received AAV9.IGF-1Ea (Figure 3d) and a reduction in the number of segments with altered motility (Figure 3e). Moreover, mice overexpressing IGF-1Ea showed fewer akinetic segments compared to I/R and I/R+GFP control groups (Figure 3g). In support of the functional data, levels of the heart failure marker Acta1 were significantly reduced with AAV9.IGF-1Ea treatment (Figure 3f). In agreement with our evidence that initial infarct size was not changed, no significant difference was observed between the different groups for either wall score motion index or individual segment assessment at 3 days post-I/R (Supplementary Table S1). Both the wall score motion index and the contraction assessment indicate that AAV9.IGF-1Ea treatment improves the overall contractile function of the heart.

### AAV9.IGF-1Ea gene transfer reduces infarct size and cardiac fibrosis

**Figure 4:**
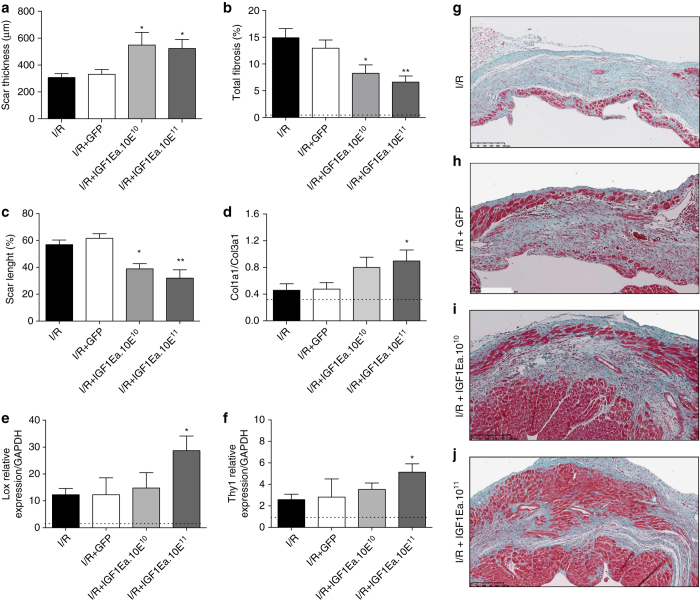
AAV9.IGF-1Ea treatment reduces infarct expansion and myocardial remodelling in AAV9.IGF-1Ea treated mice. (**a**) Scar thickness, (**b**) total fibrosis and (**c**) scar lenght were analysed 28 days post-I/R using histological methods. (**d**) Col1α1/Col3α1 ratio, (**e**) Lox and (**f**) Thy1 mRNA relative levels were analysed by qRT-PCR. (**g**–**j**) Representative masson’s thricome staining of infarct regions from all four groups. (**g**) I/R group, (**h**) I/R+GFP group, (**i**) I/R+IGF1Ea.10^10^ and (**j**) I/R+IGF1Ea.10^11^. *n*= 5–12 per group. *P<0.05, **P<0.001. I/R versus all three groups. One-way ANOVA followed with Dunnett’s post-test. Scale bar, 250 μm. Results are expressed as mean fold induction ±s.e.m. over the values of uninjured hearts (dashed line).

We performed histological analysis to determine infarct size, infarct expansion and scar thickness after AAV9.IGF-1Ea treatment. Improved cardiac function in the AAV9.IGF-1Ea groups was accompanied by thicker scars when compared with the no viral treatment (I/R) and I/R+GFP control groups (Figure 4a), along with a reduction in total heart fibrosis (Figure 4b). Scar length was also diminished in the IGF-1Ea-treated groups (Figure 4c), suggesting reduced infarct expansion. Functional and histological findings were confirmed at molecular level by monitoring LV fibrosis markers such as collagen 1 (Col1α1), collagen 3 (Col1α3), lysyl oxidase (Lox) and Thy1/CD90. Measured 28 days after IGF-1Ea cardiac gene transfer, the Col1α1/Col1α3 mRNA ratio was increased in the AAV9.IGF-1Ea groups (Figure 4d and Supplementary Figure S3A), indicating a difference in extracellular matrix composition. Higher levels of Lox were detected. Lox acts as crosslink molecule between collagen and elastin molecules into mature fibres (Figure 4e) potentially strengthening the scar. As a measurement of fibroblast proliferation, we quantified Thy1/CD90 levels, which were increased in the AA9.IGF-1Ea group (Figure 4f). No changes were observed in the macrophage marker CD68 (Supplementary Figure S3B).

**Figure 5:**
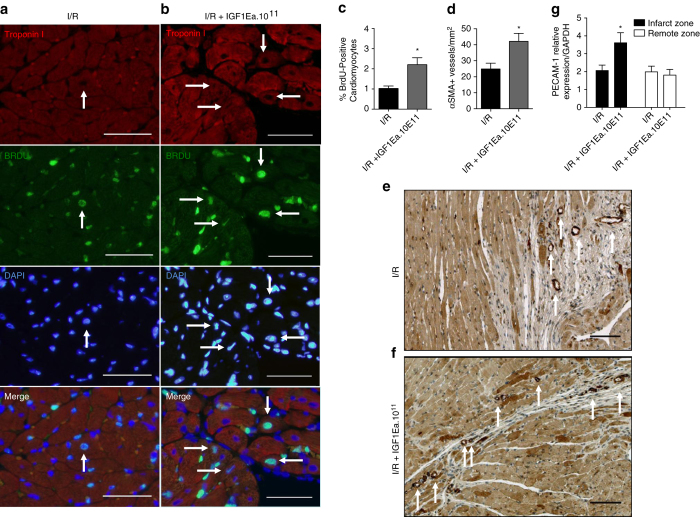
AAV9.IGF-1Ea increases the number of BrdU-positive cardiomyocytes in mice 28 post-I/R. (**a**, **b**) Representative confocal images of paraffin heart sections 28 days post-I/R. (**a**) I/R (no virus) and (**b**) AAV9.IGF-1Ea 3.5×10^11^ groups. Sections were stained with DAPI (blue, nuclei), anti-BrdU (green) and cardiac troponin I (red, cardiomyocytes). Scale bar, 100 μm. White arrows indicate BrdU-positive nuclei. (**c**) Quantification of BrdU+ cardiomyocytes after *in vivo* labeling. *n*=3–6. (**d**) αSMA immunohistochemistry of (**e**) no virus group (I/R) and (**f**) AAV9.IGF-1Ea 3.5×10^11^ treated hearts after I/R. Scale bar, 100 μm. (**g**) Platelet endothelial cell adhesion molecule-1 (PECAM-1) mRNA was analysed by qRT-PCR in the ischemic and remote myocardium. *n*=4–6 per group. Two-tailed Student’s *t*-test was performed to compare I/R versus AAV9.IGF-1Ea 3.5×10^11^ 28 days after myocardial infarction (MI). *P<0.05. Mean values ±s.e.m. are shown.

Interestingly, scars of AAV9.IGF-1Ea treated group were characterised by higher cardiomyocyte content (Figure 4g–j). To assess the generation of new myocardial tissue in the infarcted hearts, mice were given BrdU for 28 days to visualise cumulative DNA synthesis. Consistent with observations in cardiac-specific IGF-1Ea transgenic mice,^16^ the I/R+AAV9.IGF-1Ea.10^11^ group moderately increased percentage of BrdU-positive cardiomycytes at 28 days post-I/R (Figure 5a–c). Phospho-H3 staining, a mitosis marker, did not reveal any significant difference between the groups at 28 days (Supplementary Figure S4), suggesting a lack of on-going proliferation at that stage. We also assessed whether IGF-1Ea gene transfer promoted enhanced vascularisation for proper blood supply to the scars. Indeed, αSMA immunohistochemistry showed an increase in arterioles in the AAV9.IGF-1Ea-treated groups with the highest titre (Figure 5d–f). Histological findings were confirmed by quantifying the mRNA levels of PECAM-1 (CD31) as marker vascular growth (Figure 5g). The greatest difference was obtained with the I/R+AAV9.IGF-1Ea.10^11^ thus the higher viral titre was used in preceding experiments. As no significant differences were observed between the no viral treatment group (I/R) and the I/R+GFP control groups, only I/R is presented.

### AAV9.IGF-1Ea treatment promotes cardiac Akt-mediated signalling

**Figure 6:**
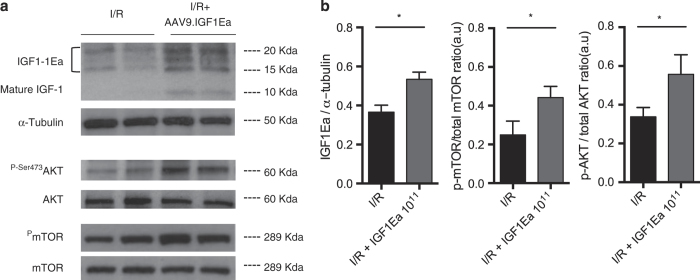
IGF-1Ea gene transfer activates the Akt signalling pathway. (**a**) Representative western blot images of protein extracts from the remote myocardium of I/R (untreated group) and AAV9.IGF-1Ea treated group 28 days post-I/R. (**b**) Western blot quantification of IGF-1Ea, p-Akt, and mTOR 28 days after cardiac transfer. *n*= 6–10 per group. Two-tailed Student’s *t*-test was performed to compare I/R versus AAV9.IGF-1Ea 3.5×10^11^ 28 days after MI. *P<0.05. Mean values ±s.e.m. are shown.

The main effects of IGF-1 are achieved through the Akt signalling pathway. In the heart, IGF-1 mediates Akt phosphorylation on the serine-473 residue, which indicates Akt activation.^24^ AAV9.IGF-1Ea treatment increased IGF-1 protein content in the heart by 1.5-fold when compared with the I/R group. At 28 days post-I/R, mice treated with AAV9.IGF-1Ea exhibited a 1.7-fold increase in ^P-Ser473^Akt. Phosphorylation of mTOR, downstream of Akt, was also increased by 1.8-fold after I/R in the AAV9.IGF-1Ea-treated groups (Figure 6a,b).

## Discussion

In this study, we have demonstrated for the first time that a single intravenous injection of AAV9.IGF-1Ea after ischemic injury induced improvements in LV cardiac function post-I/R through partial recovery of LV contraction and quality motion, reducing total fibrosis and infarct expansion limiting adverse remodelling. Interestingly, analysis of the main extracellular matrix components, collagen 1 and collagen 3, showed a skewing in favour of collagen synthesis which may confer different mechanical and physical properties to the infarct. As with previous models of IGF-1 administration, the AAV9 virus was able to induce an increase in the formation of new capillaries.^25^ Although the salutary effects of direct intramyocardial injection of IGF-1 expression vectors has been previously documented.^25–27^ This is the first report of sustained effective improvement by virally encoded IGF-1Ea propeptide in a more therapeutically relevant delivery mode.

Over the years, the use of AAVs in preclinical^28–30^ and clinical studies^31,32^ has positioned AAVs as the vector of choice for cardiac gene transfer. This is mainly due to the diverse tissue tropism determined by the capsid serotype, the lack of pathogenicity, low immunogenicity and robust gene expression achieved with this system.^19^ We also show that single administration of the AAV9 virus provided robust and early onset of IGF-1Ea expression in the heart, with the cTNT promoter proving sufficient to achieve therapeutic IGF-1Ea transcription after single systemic injection. AAV9-mediated luciferase gene expression was also detected in the liver at much lower levels, which overrode the cardiac specificity of the cTNT promoter.^22,29,33^ However, this was not paralleled by an increase in IGF-1Ea mRNA levels in the liver after AAV9.IGF-1Ea treatment, indicating that IGF-1Ea overexpression was cardiac-specific. Notably, in this study, we show for the first time that the IGF-1Ea propeptide can be successfully delivered and overexpressed in the heart by Gene Therapy. The Ea moiety in the IGF-1Ea propeptide, which is efficiently cleaved, anchors IGF-1 to the extracellular matrix in other tissues preventing its clearance and reducing off-target effects.^23^ This feature dampens the risks associated with elevated systemic IGF-1 levels such as hypotension^34^ and hypoglycaemia.^35^

We have previously shown that cardiac overexpression of IGF-1Ea in transgenic mice resulted in repression of the pro-inflammatory cytokines interleukin (IL)-1β and IL-6, while the anti-inflammatory IL-4 and IL-10 showed higher expression levels when compared with wild-type (WT) animals after cardiotoxin injury. We found that IGF-1Ea decreased the number of apoptotic cells at the injured site, which is at least in part attributable to the induction of UCP-1, metallothionine 2 and the cardioprotective cytokine adiponectin^16^ reducing infarct size.

The diverse functions of IGF-1 are mediated through the same receptor (IGF-1R), which upon activation leads to the recruitment of other substrates that in turn activate different signal pathways.^36,37^ Upon IGF-1R activation, active PI3-kinase phosphorylates inositol phospholipids.^38,39^ This phosphorylation is required to induce several downstream targets such as Akt, which promotes protein synthesis and cell survival, among other functions. Although BrdU labelling showed increased percentage of positive cardiomyocites in AAV9.IGF-1Ea-treated group, no differences were observed with Phospo-H3 staining at 28 days. IGF-1Ea also induces the expression of the calcineurin splicing variant CnAβ.^40^ Interestingly, the effects of AAV9.IGF-1Ea are reminiscent of those of CnAβ1 overexpression, which results in Akt activation, improved vascularisation of the infarct region and reduced infarct expansion leading to reduced remodelling and improved cardiac function post-infarction.^41,42^

In summary, this study demonstrates that intravenous delivery of AAV9.IGF-1Ea is effective in improving post I/R cardiac function, preventing infarct expansion and dilation. This is attributed to the combined effects of a cardiotropic gene delivery vehicle, AAV9 and the pleiotropic action of IGF-1Ea in the heart. The escalating incidence of acute cardiovascular disease leading to heart failure underscores the increasingly urgent need for improved therapeutic strategies to limit cardiac tissue damage and improve functional outcomes in translational settings. From a clinical standpoint, intravenous injection is the least invasive mode of delivery for cardiac Gene Therapy, although a higher efficiency would be achieved by delivering the AVV9.IGF-1Ea vector by intracoronary infusion during cardiac catheterisation in patients. These results provide a rationale to conduct additional studies in larger animals for translation to clinical application.

## Materials and Methods

### Viral vector plasmids, production and purification

Self-complementary adeno-associated plasmid containing the IGF-1Ea cDNA was generated from a pA-cTNT-Luciferase plasmid kindly provided by Juan Bernal, CNIC, Madrid, Spain. Mus muculus IGF-1Ea consensus sequence was cloned into pA-cTNT-Luciferase backbone and construct integrity was confirmed by sequencing. AAV vectors were produced and purified by Penn Vector Core (University of Pennsylvania) as described.^43^ The vector was subjected to vector genome titrations, sodium dodecyl sulfate-polyacrylamide gel electrophoresis analysis for particle purity, and transgene expression analysis in HL-1 cells.

### Cardiac injury model

Myocardial infarction was induced by ischemia-reperfusion (I/R) in C57BL/6 male mice, 8 to 12 weeks old, obtained from Charles River. The infarcts were performed by ligation of the left coronary artery for 45 min followed by reperfusion of the artery. Surgeries were performed under general anesthesia with 3–3.5% sevoflurane and mechanical ventilation was provided during the procedure. The mortality rate in the first 24 h post-I/R was 38%; no mortality was found afterwards. Mice received analgesic treatment with buprenorphine (0.3 mg/kg, subcutaneously) after surgery. 1 mg/ml of bromodeoxyuridine (BrdU), was administered in the water of all four groups and renewed every 2 days.

### *In vivo* gene transfer

Five hours after I/R, mice were anaesthetised with 1.5–2.5% sevoflurane and 50 μl of concentrated AAV vector (in phosphate-buffered saline) was injected intravenously through the left femoral vein using a 29-gauge insulin syringe. AAV9 administration was performed as follows. One group received 3.5×10^11^ genome copies (GC) of AAV9.GFP as a AAV control. For the IGF-1Ea gene transfer, two different viral titers were administered, 3.5×10^10^ GC and 3.5×10^11^ GC of AAV9.IGF-1Ea.

### Detection of serum IGF-1 protein

The sera from the four groups were utilised for measurements of total IGF-1 using a commercially available ELISA kit specific for rodent IGF-1 (Peprotech, London, UK, 900-K170). Calculations of IGF-1 content were based on a standard curve generated from recombinant mouse IGF-1. Total rodent IGF-1 was detected according to the manufacturer’s instruction.

### Echocardiographic analysis

Transthoracic echocardiography and analysis was blindly performed at baseline, 3 and 28 days post-I/R injury in anaesthetised mice to evaluate LV systolic function, chamber dimensions, wall thickness, infarct size and wall motion. All measurements were carried out in accordance with the recommendations for chamber quantification from the American Society of Echocardiography in conjunction with the European Association of Echocardiography.^41,44^ Left ventricle wall motion score index was calculated in order to assess global and regional cardiac function by a 12-based segment model, considering parasternal two-dimensional short and long axis views at 3 levels (base, middle and apex). In each level, the left ventricle was divided in 4 segments (anterior, lateral, inferior and septal) and each segment was scored according its severity in terms of contraction as 1 (normal), 2 (hypokinetic), 3 (akinetic), 4 (dyskinetic) and 5 (aneurysmal).^44^ Number of segments affected is calculated as the number of segments with abnormal contractility out of the 12 segments of the heart (Score>1). Cardiac score is the sum of the severity score of each segment. The colour-coded heart quantification is calculated as the average score of the same segment (anterior, lateral, inferior and septal from base, middle and apex) throughout all samples. Wall motion score index defined as the ratio of the sum of scored individual segment over the total number of segments evaluated. Infarct size was also estimated considering the mean of scored individual segments. For this study, 140 mice underwent I/R with a mortality rate of 38%. An exclusion criterion was pre-established taking into account that both large and small infarct influence the outcome depending on which group could randomly be allocated. Therefore, homogeneous infarct sizes were selected according to the following exclusion criteria: Only mice that presented two or more akinetic cardiac segments plus a LV ejection fraction below 45% at 3 days post-surgery were selected for the study. After applying the exclusion criteria, mice were randomly allocated to form the four groups. Further description of the echocardiographic methods can be found in Supplementary Materials.

### Bioluminescence analysis

Mice were anaesthetised with 1.5–2% isoflurane in oxygen. D‐luciferin (Promega, Madison, WI, USA, E1601) at 100 mg/kg in saline was administered to mice by intraperitoneal injection. 5–10 min after D‐luciferin administration, all mice were imaged in supine position using a Xenogen IVIS100 imaging system (Caliper Life Sciences, Hopkinton, MA, USA). Organs were immersed in D-luciferin for 1 min and bioluminesce was imaged immediately after using the IVIS 100 system. Photons emitted from the mice were collected and integrated for 1 min. Images were processed using Living Image software (Caliper Life Sciences). Mean luminescence intensities (photons per s cm^2^ sr) were measured from the regions of interest over the mouse hearts.

### Histology, immunohistochemistry and immunofluorecesce

Scar length was determined in Masson’s trichrome-stained sections using the midline method, which best correlates with functional measurements.^45^ In this method, the infarct length is measured as the length of the midline of the infarcted wall, in which 50% of the wall thickness is composed of scar tissue. Scar length represents the percentage of infarct length with respect to the length of the whole LV circumference. Total fibrosis was quantified by ImageJ (NIH, Bethesda, MD, USA) in whole heart images obtained at the middle section. Further description of Masson’s, immunohistochemistry and immunofluorescence staining can be found in Supplementary Material.

### RNA isolation and quantitative reverse-transcriptase PCR

After sacrificing the mice, the hearts were perfused with phosphate-buffered saline, and samples from the infarct region, border zone and remote myocardium were harvested and snap-frozen in liquid nitrogen. Total RNA was isolated using the RNeasy kit from Qiagen, with DNAse digestion on the column. Quantitative reverse transcription PCR (qRT-PCR) data were analysed using the LinReg (Amsterdam, Netherlands) software in order to estimate the efficiency rates and the Ct values.^46^

### Western blot

Western blot was performed using the following primary antibodies anti-phospho-Akt-Ser473 (Cell Signaling, Danvers, MA, USA), anti-Akt (Cell Signaling), anti-phospho mTOR- Ser 2448 (Cell Signaling), anti-mammalian target of rapamycin (mTOR; Cell Signaling), IGF-1 (Sigma, Dorset, UK) and α-Tubulin (Sigma) as previously described.^47,48^ Brightness and contrast were linearly adjusted using power point.

### Statistics

Data are presented as mean±s.e.m. In addition, a two-way analysis of variance (ANOVA) with repeated measures followed by Bonferroni post-test was applied to compare mice at 3 versus 28 days post-I/R. Group differences in qRT-PCR and histological quantifications were analysed by one-way ANOVA followed by Dunnett’s post-test to compare with untreated mice. Student’s *t*-test or two-way ANOVA, followed by Bonferroni’s post-test was performed to quantify vessels and western blot analysis. G*Power 3.1 (Heinrich-Heine-Universität Düsseldorf, http://www.gpower.hhu.de) software was used to estimate sample size of each group after surgery with a 95% confidence level and 5% margin of error. Data were analysed with GraphPad-Prism 5.0 (Graphpad Software, www.graphpad.com), and differences were considered statistically significant at *P*<0.05. Further description of the statistical analysis can be found in Supplementary Material.

### Ethical Approval

All mouse procedures were approved by the Imperial College London Ethical Committee and were in accordance with national and international regulations (UK Home Office Project License 70/7589).

## References

[bib1] Go, A. S. et al. Heart disease and stroke statistics-2014 update: a report from the American Heart Association. Circulation 129, e28–e292 (2014). 24352519 10.1161/01.cir.0000441139.02102.80 PMC5408159

[bib2] Lee, D. S. et al. A systematic assessment of causes of death after heart failure onset in the community: impact of age at death, time period, and left ventricular systolic dysfunction. Circ. Heart Fail. 4, 36–43 (2011). 21071547 10.1161/CIRCHEARTFAILURE.110.957480 PMC3243964

[bib3] Yancy, C. W. et al. 2013 ACCF/AHA guideline for the management of heart failure: A report of the american college of cardiology foundation/american heart association task force on practice guidelines. Circulation 128, 240–327 (2013). 10.1161/CIR.0b013e31829e8776 23741058

[bib4] Deb, S. et al. Coronary artery bypass graft surgery versus percutaneous interventions in coronary revascularization. JAMA 310, 2086 (2013). 24240936 10.1001/jama.2013.281718

[bib5] Zweier, J. L. Measurement of superoxide-derived free radicals in the reperfused heart. Evidence for a free radical mechanism of reperfusion injury. J. Biol. Chem. 263, 1353–1357 (1988). 2826476

[bib6] Rotwein, P. , Pollock, K. M. , Didier, D. K. & Krivi, G. G. Organization and sequence of the human insulin-like growth factor I gene. Alternative RNA processing produces two insulin-like growth factor I precursor peptides. J. Biol. Chem. 261, 4828–4832 (1986). 2937782

[bib7] Frindik, J. P. & Kemp, S. F. Managing idiopathic short stature: role of somatropin (rDNA origin) for injection. Biologics 4, 147–155 (2010). 20631818 10.2147/btt.s6363 PMC2898102

[bib8] Laron, Z. Insulin-like growth factor 1 (IGF-1): a growth hormone. Mol. Pathol. 54, 311–316 (2001). 11577173 10.1136/mp.54.5.311 PMC1187088

[bib9] D’Ercole, A. J. , Stiles, A. D. & Underwood, L. E. Tissue concentrations of somatomedin C: further evidence for multiple sites of synthesis and paracrine or autocrine mechanisms of action. Proc. Natl Acad. Sci. USA 81, 935–939 (1984). 6583688 10.1073/pnas.81.3.935 PMC344954

[bib10] Musarò, a. et al. Localized Igf-1 transgene expression sustains hypertrophy and regeneration in senescent skeletal muscle. Nat. Genet. 27, 195–200 (2001). 11175789 10.1038/84839

[bib11] Hill, M. & Goldspink, G. Expression and splicing of the insulin-like growth factor gene in rodent muscle is associated with muscle satellite (stem) cell activation following local tissue damage. J. Physiol. 549, 409–418 (2003). 12692175 10.1113/jphysiol.2002.035832 PMC2342958

[bib12] Barton-Davis, E. R. , Shoturma, D. I. , Musaro, A. , Rosenthal, N. & Sweeney, H. L. Viral mediated expression of insulin-like growth factor I blocks the aging-related loss of skeletal muscle function. Proc. Natl Acad. Sci. USA 95, 15603–15607 (1998). 9861016 10.1073/pnas.95.26.15603 PMC28090

[bib13] Pelosi, L. et al. Local expression of IGF-1 accelerates muscle regeneration by rapidly modulating inflammatory cytokines and chemokines. FASEB J. 21, 1393–1402 (2007). 17264161 10.1096/fj.06-7690com

[bib14] Tonkin, J. et al. Monocyte/macrophage-derived IGF-1 orchestrates murine skeletal muscle regeneration and modulates autocrine polarization. Mol. Ther. 23, 1189–1200 (2015). 25896247 10.1038/mt.2015.66 PMC4817788

[bib15] Gallego-Colon, E. et al. Cardiac-restricted IGF-1Ea overexpression reduces the early accumulation of inflammatory myeloid cells and mediates expression of extracellular matrix remodeling genes after myocardial infarction. Madiat. Inflamm. 2015, 484357 (2015). 10.1155/2015/484357 PMC4605352 26491228

[bib16] Santini, M. P. et al. Enhancing repair of the mammalian heart. Circ. Res. 100, 1732–1740 (2007). 17525368 10.1161/CIRCRESAHA.107.148791 PMC3227120

[bib17] Gregorevic, P. et al. Systemic delivery of genes to striated muscles using adeno-associated viral vectors. Nat. Med. 10, 828–834 (2004). 15273747 10.1038/nm1085 PMC1365046

[bib18] Wang, Z. et al. Adeno-associated virus serotype 8 efficiently delivers genes to muscle and heart. Nat. Biotechnol. 23, 321–328 (2005). 15735640 10.1038/nbt1073

[bib19] Weitzman, M. D. & Linden, R. M. Adeno-associated virus biology. Methods Mol. Biol. 807, 1–23 (2011). 22034024 10.1007/978-1-61779-370-7_1

[bib20] Pacak, C. a. et al. Recombinant adeno-associated virus serotype 9 leads to preferential cardiac transduction in vivo. Circ. Res. 99, e3–e9 (2006). 16873720 10.1161/01.RES.0000237661.18885.f6

[bib21] Bostick, B. , Ghosh, A. , Yue, Y. , Long, C. & Duan, D. Systemic AAV-9 transduction in mice is influenced by animal age but not by the route of administration. Gene Ther. 14, 1605–1609 (2007). 17898796 10.1038/sj.gt.3303029

[bib22] Prasad, K.-M. R. , Xu, Y. , Yang, Z. , Acton, S. T. & French, B. A. Robust cardiomyocyte-specific gene expression following systemic injection of AAV: in vivo gene delivery follows a Poisson distribution. Gene Ther. 18, 43–52 (2011). 20703310 10.1038/gt.2010.105 PMC2988989

[bib23] Hede, M. S. et al. E-peptides control bioavailability of IGF-1. PLoS ONE 7, e51152 (2012). 23251442 10.1371/journal.pone.0051152 PMC3519493

[bib24] Vinciguerra, M. et al. mIGF-1/JNK1/SirT1 signaling confers protection against oxidative stress in the heart. Aging Cell 11, 139–149 (2012). 22051242 10.1111/j.1474-9726.2011.00766.x

[bib25] Dobrucki, L. W. et al. Analysis of angiogenesis induced by local IGF-1 expression after myocardial infarction using microSPECT-CT imaging. J. Mol. Cell Cardiol. 48, 1071–1079 (2010). 19850049 10.1016/j.yjmcc.2009.10.008 PMC2866767

[bib26] Davis, M. E. et al. Local myocardial insulin-like growth factor 1 (IGF-1) delivery with biotinylated peptide nanofibers improves cell therapy for myocardial infarction. Proc. Natl Acad. Sci. USA 103, 8155–8160 (2006). 16698918 10.1073/pnas.0602877103 PMC1472445

[bib27] Khan, R. S. et al. Targeting extracellular DNA to deliver IGF-1 to the injured heart. Sci. Rep. 4, 4257 (2014). 24604065 10.1038/srep04257 PMC3945489

[bib28] Konishi, M. , Kawamoto, K. , Izumikawa, M. , Kuriyama, H. & Yamashita, T. Gene transfer into guinea pig cochlea using adeno-associated virus vectors. J. Gene Med. 10, 610–618 (2008). 18338819 10.1002/jgm.1189

[bib29] Kaspar, B. K. et al. Myocardial gene transfer and long-term expression following intracoronary delivery of adeno-associated virus. J. Gene Med. 7, 316–324 (2005). 15515115 10.1002/jgm.665

[bib30] Müller, O. J. et al. Improved cardiac gene transfer by transcriptional and transductional targeting of adeno-associated viral vectors. Cardiovasc. Res. 70, 70–78 (2006). 16448634 10.1016/j.cardiores.2005.12.017

[bib31] Zacchigna, S. , Zentilin, L. & Giacca, M. Adeno-associated virus vectors as therapeutic and investigational tools in the cardiovascular system. Circ. Res. 114, 1827–1846 (2014). 24855205 10.1161/CIRCRESAHA.114.302331

[bib32] Chacon-Camacho, O. F. Review and update on the molecular basis of Leber congenital amaurosis. World J. Clin. Cases 3, 112 (2015). 25685757 10.12998/wjcc.v3.i2.112 PMC4317604

[bib33] Fang, H. et al. Comparison of adeno-associated virus serotypes and delivery for cardiac gene transfer. Hum. Gene Ther. Methods 23, 234–241 (2013). 10.1089/hgtb.2012.105 PMC3555516 22966786

[bib34] Cheng, P.-W. et al. Involvement of two distinct signalling pathways in IGF-1-mediated central control of hypotensive effects in normotensive and hypertensive rats. Acta Physiol. 212, 28–38 (2014). 10.1111/apha.12340 24995704

[bib35] Kovacs, G. T. et al. Hypoglycemic effects of insulin-like growth factor-1 in experimental uremia: Can concomitant growth hormone administration prevent this effect? Horm. Res. 51, 193–200 (1999). 10474022 10.1159/000023357

[bib36] Butler, A. A. et al. Insulin-like growth factor-I receptor signal transduction: at the interface between physiology and cell biology. Comp. Biochem. Physiol. B Biochem. Mol. Biol. 121, p 19–26 (1998). 9972281 10.1016/s0305-0491(98)10106-2

[bib37] Coolican, S. A. , Samuel, D. S. , Ewton, D. Z. , McWade, F. J. & Florini, J. R. The mitogenic and myogenic actions of insulin-like growth factors utilize distinct signaling pathways. J. Biol. Chem. 272, 6653–6662 (1997). 9045696 10.1074/jbc.272.10.6653

[bib38] Fujio, Y. , Nguyen, T. , Wencker, D. , Kitsis, R. N. & Walsh, K. Akt promotes survival of cardiomyocytes in vitro and protects against ischemia-reperfusion injury in mouse heart. Circulation 101, 660–667 (2000). 10673259 10.1161/01.cir.101.6.660 PMC3627349

[bib39] Rommel, C. et al. Mediation of IGF-1-induced skeletal myotube hypertrophy by PI(3)K/Akt/mTOR and PI(3)K/Akt/GSK3 pathways. Nat. Cell Biol. 3, 1009–1013 (2001). 11715022 10.1038/ncb1101-1009

[bib40] Lara-Pezzi, E. et al. A naturally occurring calcineurin variant inhibits FoxO activity and enhances skeletal muscle regeneration. J. Cell Biol. 179, 1205–1218 (2007). 18086917 10.1083/jcb.200704179 PMC2140042

[bib41] López-Olañeta, M. M. et al. Induction of the calcineurin variant CnAβ1 after myocardial infarction reduces post-infarction ventricular remodelling by promoting infarct vascularization. Cardiovasc. Res. 102, 396–406 (2014). 24667850 10.1093/cvr/cvu068

[bib42] Santini, M. P. et al. IGF-1Ea induces vessel formation after injury and mediates bone marrow and heart cross-talk through the expression of specific cytokines. Biochem. Biophys. Res. Commun. 410, 201–207 (2011). 21621517 10.1016/j.bbrc.2011.05.081

[bib43] Gao, G. et al. Biology of AAV serotype vectors in liver-directed gene transfer to nonhuman primates. Mol. Ther. 13, 77–87 (2006). 16219492 10.1016/j.ymthe.2005.08.017

[bib44] Lang, R. M. et al. Recommendations for chamber quantification: A report from the American Society of Echocardiography’s guidelines and standards committee and the Chamber Quantification Writing Group, developed in conjunction with the European Association of Echocardiograph. J. Am. Soc. Echocardiogr. 18, 1440–1463 (2005). 16376782 10.1016/j.echo.2005.10.005

[bib45] Takagawa, J. et al. Myocardial infarct size measurement in the mouse chronic infarction model: comparison of area- and length-based approaches. J. Appl. Physiol. 102, 2104–2111 (2007). 17347379 10.1152/japplphysiol.00033.2007 PMC2675697

[bib46] Ruijter, J. M. et al. Amplification efficiency: linking baseline and bias in the analysis of quantitative PCR data. Nucleic Acids Res. 37, e45 (2009). 19237396 10.1093/nar/gkp045 PMC2665230

[bib47] Blackstock, C. D. et al. Insulin-like growth factor-1 increases synthesis of collagen type I via induction of the mRNA-binding protein LARP6 expression and binding to the 5’stem-loop of COL1a1 and COL1a2 mRNA. J. Biol. Chem. 289, 7264–7274 (2014). 24469459 10.1074/jbc.M113.518951 PMC3953245

[bib48] Panse, K. D. et al. Follistatin-like 3 mediates paracrine fibroblast activation by cardiomyocytes. J. Cardiovasc. Transl. Res. 5, 814–826 (2012). 22915069 10.1007/s12265-012-9400-9

