# Erratum: Intravenous delivery of adeno-associated virus 9-encoded IGF-1Ea propeptide improves post-infarct cardiac remodelling

**DOI:** 10.1038/npjregenmed.2017.1

**Published:** 2017-10-20

**Authors:** Enrique Gallego-Colon, Maria Villalba, Joanne Tonkin, Francisco Cruz, Juan Antonio Bernal, Luis J Jimenez-Borregureo, Michael D Schneider, Enrique Lara-Pezzi, Nadia Rosenthal

**Correction to:** *npj Regenerative Medicine* (2016) **1**, 16001; doi:10.1038/npjregenmed.2016.1; published online 9 June 2016

The following statement was missing in the Materials and Methods section: All mouse procedures were approved by the Imperial College London Ethical Committee and were in accordance with national and international regulations (UK Home Office Project License 70/7589).

A correction has been published and the statement has now been added in the HTML and PDF versions of this Article.

