# Supplementary material for: Intravenous delivery of adeno-associated virus 9-encoded IGF-1Ea propeptide improves post-infarct cardiac remodelling
Source: NPJ Regen Med. 2016 Jun 9;1:16001–. doi: 10.1038/npjregenmed.2016.1 (PMC5744701; doi:10.1038/npjregenmed.2016.1)
Supplement: Supplementary Material [file npjregenmed20161-s1.doc]

**SUPPLEMENTAL MATERIAL**

**Echocardiography**

Echocardiographic measurements were taken by a blinded operator using a high-frequency ultrasound system Vevo 2100 (VisualSonics, Inc., Toronto, ON, Canada) with a 30-MHz linear transducer and recorded images were analysed by a second blinded operator using the Vevo 2100 workstation software. Mice were anesthetized with 1-2% isoflurane in oxygen, the anesthetic flow was adjusted to maintain heart rate in approximately 450 ± 50 beats per minute. Furthermore, warmed ultrasound gel and a heating platform were used to preserve body temperature around 37 ± 0.5ºC to minimize variation among mice. Standard 2D and M-mode parasternal long and short axis (at mid-ventricle) views were obtained to assess left ventricle function and chamber dimensions. From these modes, left ventricular end-systolic volume (LVESV), left ventricular end-diastolic volume (LVEDV), left ventricular ejection fraction (LVEF), left ventricular end-systolic diameter (LVESd), left ventricular end-diastolic diameter (LVEDd) and left ventricular ejection fractional (LVEF) were acquired to perform the analysis. Left ventricle wall motion score index (WMSI) was calculated in order to assess global and regional cardiac function by a 12-based segment model, considering parasternal 2D short and long axis views at 3 levels (base, middle and apex). In each level, the left ventricle was divided in 4 segments (anterior, lateral, inferior and septal) and each segment was scored according its severity in terms of contraction as 1 (normal), 2 (hypokinetic), 3 (akinetic), 4 (dyskinetic) and 5 (aneurysmal)^22^. Number of segments affected is calculated as the number of segments with abnormal contractility out of the 12 segments of the heart (Score > 1). Cardiac score is the sum of the severity score of each segment. The color-coded heart quantification is calculated as the average score of the same segment (anterior, lateral, inferior and septal from base, middle and apex) throughout all samples. WMSI defined as the ratio of the sum of scored individual segment over the total number of segments evaluated. Infarct size was also estimated considering the mean of scored individual segments. For this study, 140 mice underwent I/R with a mortality rate of 38%. An exclusion criterion was pre-established taking into account that both large and small infarct influence the outcome depending on which group could randomly be allocated. Therefore, homogeneous infarct sizes were selected according to the following exclusion criteria: Only mice that presented two or more akinetic cardiac segments plus a LV ejection fraction below 45% at 3 days post-surgery were selected for the study. After applying the exclusion criteria, mice were randomly allocated to form the four groups. Further description of the echocardiographic methods can be found in supplementary materials.

**Histology, immunohistochemistry and immunofluorecesce**

After scarifying the mice, KCl-arrested-perfused hearts samples were fixed in paraformaldehyde (4% in PBS) for 48h, washed in PBS, dehydrated, and included in paraffin. Five-micron thick sections were stained following Masson’s trichrome protocol. Paraffin sections were stained with Celestine Blue for 5 minutes, washed in tap water and incubated in haemotoxylin for 5 minutes, washed in tap water very well for 5 minutes. Slides were incubated with Acid Fucsin for 5 minutes, Rinsed quickly in distilled water, differentiated in Phosphomolybdic acid (1%) for 3-5 minutes.Rinsed briefly in water. Stained in Methyl Blue for 2-3 minutes (connective tissue stain) Slides were washed briefly with distilled water and dehydrated in ascending series of Methanol, cleared in xylene and mounted in DPX (all from VWR).

For immunohistochemistry, prior to immunoperoxidase staining, 5μm thick paraffin wax sections were dewaxed and rehydrated in to water, washed in phosphate buffered saline (PBS) for 5 minutes. Then the slides were immersed in 0.1M citrate buffer (pH 6) and microwave for 10 minutes before blocking for endogenous peroxidases using 0.3% hydrogen peroxide in PBS for 10 minutes. Sections were washed 3x5 minutes each in PBS and blocked with 3% bovine serum albumin (W/V) (BSA) in PBS for 30 minutes. Sections were incubated separately overnight with primary antibodies. The primary antibodies used were, IGF-1 (Sigma, UK. Cat. I-8773) antibody 1:100 dilution, anti-α-smooth muscle actin (Abcam, UK. Cat. Ab5694) 1:400 dilution, and for Luciferease (Promega, USA. Cat. G7451) 1:300 dilution was used. Negative control consisted of 3% BSA in PBS. Primary antibodies was then removed by washing the sections 3 times in PBS followed by a second layer of Biotinylated goat anti-goat immunoglobulins (Vector laboratories, UK. Cat. I-5000) diluted 1/250 in PBS for 1hour. Sections were then washed 3 times in PBS before 1-hour incubation with Avidin-Biotin (Vector laboratories, UK. Cat. PK-4000)). Reactivity was detected using diaminobenzidine tetrahydrochloride (DAB tablets-25mg/ml) (Sigma, UK Cat. D4293) and hydrogen peroxide (0.01% W/V). Sections were then washed 3 times in PBS before 1-hour incubation with Avidin-Biotin Complex ABC-Vector laboratories). Reactivity was detected using diaminobenzidine tetrahydrochloride (DAB tablets- Sigma) (25mg/ml) and hydrogen peroxide (0.01% W/V). Sections were then counter stained with haematoxylin and viewed on Ziess Axioskop microscope. Vessels were quantified by anti-α-smooth muscle actin (αSMA) immunohistochemistry. The number of vessels was determined in the infarct region divided by the infarct area. Images were digitalised using a NanoZommer (Hamamatsu) and further quantified using ImageJ (NIH, USA).

For immunofluorescence, 5μm thick paraffin wax sections were dewaxed and rehydrated in to water, washed in phosphate buffered saline (PBS) for 5 minutes. Then the slides were immersed in 0.1M citrate buffer (pH 6) and microwave for 10 minutes before blocking for endogenous peroxidases using 0.3% hydrogen peroxide in PBS for 10 minutes. Sections were washed 3x5 minutes each in PBS-Triton 0.3% and blocked with 3% bovine serum albumin (W/V) (BSA), 20mM MgCl_2_, 0.3% Tween, 5% goat serum in PBS for 1 hours at RT. Samples were block with Avidin/Biotin blocking kit (Vector Labs, USA, SP-2001). Sections were washed 3x5 minutes each in PBS-Triton 0.3% and incubated O/N at 4°C. For BrdU and troponin T immunofluorescence, mouse anti-BrdU 1:30 dilution (BD Bioscience, USA, Cat. 347580). Rabbit Anti-cardiac troponin I 1:100 dilution (Abcam, UK, Cat. ab 47003). Sections were washed 3x5 minutes each in PBS-Triton 0.3% and incubated O/N at 4°C. The following day slides were incubated a biotinylated anti-rabbit secondary antibody 1:100 (Jackson ImmunoResearch, USA, Cat.111-066-003) for 1 hour at room temperature. Finally, slides were incubated with anti-mouse streptavidin-Alexa 488 1:200 (ThermoFisher, USA, Cat. A-11029) and anti-biotin Streptavidin-Cy3 1:200 (Jackson ImmunoResearch, USA, Cat. 016-160-084). Sections were washed 3x5 minutes each in PBS with a DAPI 1:1000. Slides double-stained by anti-BrdU and troponin I antibody and assessed by Zeiss LSM Confocal Microscope System.

**RNA isolation and quantitative reverse-transcriptase polymerase chain reaction**

100 ng of total RNA using random hexamers and high capacity cDNA Reverse Transcription kit (Applied Biosystems) in a 10μl reaction. Quantitative reverse-transcribed polymerase chain reaction (qRT-PCR) was carried out in an AB9700 thermocycler (Applied Biosystems) using Taqman chemistry or SYBR green (Applied Biosystems). The following Taqman probes were used: actin, alpha 1, skeletal muscle, Acta1 (Mm00808218_g1), Nppb/brain natriuretic peptide (BNP) (Mm01255770_g1), Collagen Iα1 (Mm00801666_g1), Collagen Iα3 (Mm01254476_m1), Lox (Mm00495386_m1), Thy1 (Mm00493681_m1), IGF-1Ea (transcript variant 4) (Mm00710307-m1), Luciferase (Mr03987587_mr), PECAM-1 (Mm Mm01242576_m1). Gene expression was normalized to GAPDH RNA levels quantified simultaneously using a VIC-labelled probe (4310893E, Applied Biosystems). GFP expression was quantified using SYBR green primers: FW EGFP: CCA GGA GCG CAC CAT CTT CTT. And RV EGFP: GTA GTG GTT GTC GGG CAG CAG.

**Statistics**

Data are presented as mean +/- SEM. AAV9 administration was performed 5 hours after surgery by intra-femoral vein injection and mice were sacrificed 28 days after injection. Samples were collected at 28 days for further analysis. All the data was analyzed with GraphPad-Prism 5.0 (Graphpad Software, Inc., www.graphpad.com), and differences were considered statistically significant at P <0.05. G*Power 3.1 (Heinrich-Heine-Universität Düsseldorf, http://www.gpower.hhu.de) software was used to estimate sample size of each group after surgery with a 95% confidence level and 5% margin of error.

**Figure 1:** n=3. *P<0.05, **P<0.001. One way ANOVA with Dunnett’s Multiple Comparison test. I/R group (no viral treatment group) as control group.

**Figure 2:** (B) n=5-12 per group. (C) n=6-12 per group. *P<0.05, **P<0.001. One-way ANOVA, Dunnett’s Multiple Comparison test with the I/R group (no viral treatment group).

**Figure 3:** n=7-12 per group**.** *P<0.05 I/R vs I/R + GFP group, I/R + IGF1Ea.10^10^ and I/R + IGF1Ea.10^11^ groups. One-way ANOVA with Dunnett's Multiple Comparison post-test. †P<0.05. Two-way ANOVA to compare the 4 groups with Bonferroni post-tests. ‡ P<0.05. Two-way ANOVA to compare 3 days vs. 28 days with Bonferroni post-tests..

**Figure 4:** Results are expressed as mean fold induction +/- SEM over the values of uninjured hearts (dashed line). n= 5-12 per group. * P<0.05, ** P<0.001. I/R vs. all three groups. One-way ANOVA followed with Dunnett’s post-test. Scale bar, 250 μm.

**Figure 5:** n=4-6 per group. Two-tailed Student’s *t*-test was performed to compare I/R vs AAV9.IGF-1Ea 3.5x10^11^ 28 days after MI. *p<0.05.

**Figure 6:** n= 6-10 per group. Two-tailed Student’s *t*-test was performed to compare I/R vs AAV9.IGF-1Ea 3.5x10^11^ 28 days after MI. *p<0.05.
