# Supplementary material for: Intravenous delivery of adeno-associated virus 9-encoded IGF-1Ea propeptide improves post-infarct cardiac remodelling
Source: NPJ Regen Med. 2016 Jun 9;1:16001–. doi: 10.1038/npjregenmed.2016.1 (PMC5744701; doi:10.1038/npjregenmed.2016.1)
Supplement: Supplementary Table 1 [file npjregenmed20161-s2.doc]

Supplementary Table 1.

|  | | **3 Days post I/R** | | | |  | |
| --- | --- | --- | --- | --- | --- | --- | --- |
|  | | **I/R** | **I/R + AAV9.GFP** | **I/R + AAV9.IGF-1Ea** | **I/R + AAV9.IGF-1Ea** |  | |
|  |  | | **3.5x10^11^ GC** | **3.5x10^10^ GC** | **3.5x10^11^ GC** |  |  |
| **LVIDD** | | 3,647±0.1249 | 3.978±0.1978 | 3.782±0.1363 | 3.757±0.1431 |  | |
| **LVIDS** | | 2,909±0.129 | 3.251±0.1334 § | 3.192±0.1345 § | 3.236±0.1563 § |  | |
| **LVEDV** | | 62,07±4,661 | 55,3±4,093 § | 58,94±7,967 § | 57,11±4,329 § |  | |
| **LVESV** | | 47,34±4,199 § | 42,53±3,303 § | 45,73±7,232 § | 44,25±4,031 § |  | |
| **LVEDA** | | 12.76±0.6272 | 13.56±0.8405 | 13.54±1.338 | 12.43±0.9266 |  | |
| **LVESA** | | 9,543±0,5581 § | 9,99±0,6829 § | 10,37±1,017 § | 9,183±0,9007 § |  | |
| **Number of**  **segments affected** | | *6.038±0.3645* | *5.4±0.6403* | *5.714±0.5759* | *4.857±0.8071* |  | |
| **Cardiac score** | | *22,77±3,03* | *21,71±4,11* | *20,71±3,15* | *21,30±4,19* |  | |
| **WSMI** | | 1,90±0,25 | 1,81±0,34 | 1.73±0.26 | 1,78±0,35 |  | |
|  | |  |  |  |  |  | |
|  | | **28 Days post I/R** | | | | | |
|  | | **I/R** | **I/R + AAV9.GFP** | **I/R + AAV9.IGF1Ea** | **I/R + AAV9.IGF1Ea** | **No** | |
|  |  | | **3.5x10^11^ GC** | **3.5x10^10^ GC** | **3.5x10^11^ GC** | **I/R** |  |
| **LVIDD** | | 4.627±0.1226 ‡§ | 4.705±0.2768 ‡§ | 4.291±0.1993 *§ | 4.016±0.1145 *† | 3.865±0.07566 | |
| **LVIDS** | | *4.022±0.1352* ‡§ | 3.62±0.2105 ‡§ | 3.183±0.1778 | 3.216±0.2105 *†§ | 2.860±0.07335 | |
| **LVEDV** | | *104.8±8.251* ‡§ | 104.1±15.72 ‡§ | 85.51±2.737§ | 72.6±3.541 † | 66.51±2.59 | |
| **LVESV** | | *80.01±5.973* ‡§ | 75.58±12.47 ‡§ | 46.36±3.986 *†§ | 39.89±3.386 *†§ | 31.74±9.21 | |
| **LVEDA** | | *17.04±0.5598* ‡§ | 17.53±1.801 ‡§ | 16.36±1.123 § | 14.07±0.8024 | 13.65±0.5122 | |
| **LVESA** | | *12.24±0.6517* ‡§ | 12.85±1.412 ‡§ | 10.21±0.9048 § | 8.491±0.6365 *† | 7.291±0.2907 | |
| **Number of**  **segments affected** | | *5.867±0.3326* | 6±1.204 | 4.571±1.077 † | 3.7±0.4955 *†‡ | 0.0±0.0 | |
| **Cardiac score** | | *23,69±3,25* | 22,50±7,50 | 18,57±5,35 * | 16,20±1,99 *†‡ | 12±0.0 | |
| **WSMI** | | 1,97±0,27 | 1,88±0,63 | 1,55±0,45 *† | 1,35±0,17 *†‡ | 1±0.0 | |
| **HW/BW** | | 7,98±2,14 | 8,11±1,93 | 7,99±1,21 | 7,71±2,87 | NE | |
| **n** | | 13 | 7 | 10 | 10 | 20 | |

**Supplementary Table 1.** Echocardiographic measurements at 3 and 28 days after ischemia/reperfusion (I/R). LVIDD, left ventricular internal diameter in diastole; LVIDS, left ventricular internal diameter in systole; LVEDV, left ventricular end-diastolic volume; LVESV, left ventricular end-systolic volume; LVEDA, left ventricular end-diastolic area; LVESA, left ventricular end-systolic area. Left ventricle wall motion was calculated to assess global and regional cardiac function by a 12-based segment model, considering 2D short axis views at 3 levels (base, middle and apex). In each level, the left ventricle was divided in 4 segments (anterior, lateral, inferior and septal) and each segment was scored according its severity in terms of contraction as: 1 (normal), 2 (hypokinetic), 3 (akinetic), 4 (dyskinetic) and 5 (aneurysmal)^22^. Number of segments affected is calculated as the number of segments, with a severity score >1, and abnormal contractility out of the 12 segments. Cardiac score is the sum of the severity score of each segment. The color-coded heart quantification is calculated as the average score of the same segment (anterior, lateral, inferior and septal from base, middle and apex) throughout all samples. Wall Motion Score Index (WMSI) is defined as the ratio of the sum of scored individual segment over the total number of segments evaluated. Infarct size was also estimated considering the mean of scored individual segments. HW/BW, heart weight/body weight. n=7-13. Mean values +/- SEM are shown.

* P<0.05 I/R vs AAV9.GFP, AAV9.IGF-1Ea groups. One-way ANOVA with Dunnett's Multiple Comparison post-test. † P<0.05. Two-way ANOVA to compare the 4 groups with Bonferroni post-tests. ‡ P<0.05. Two-way ANOVA to compare 3 days vs. 28 days with Bonferroni post-tests. § P<0.05 Two-tailed Student’s t-test was performed to compare infarcted versus no I/R groups at 3 and 28 days.
