# Supplementary material for: Intravenous delivery of adeno-associated virus 9-encoded IGF-1Ea propeptide improves post-infarct cardiac remodelling
Source: NPJ Regen Med. 2016 Jun 9;1:16001–. doi: 10.1038/npjregenmed.2016.1 (PMC5744701; doi:10.1038/npjregenmed.2016.1)
Supplement: Supplementary Information [file npjregenmed20161-s3.ppt]

## Slide 1
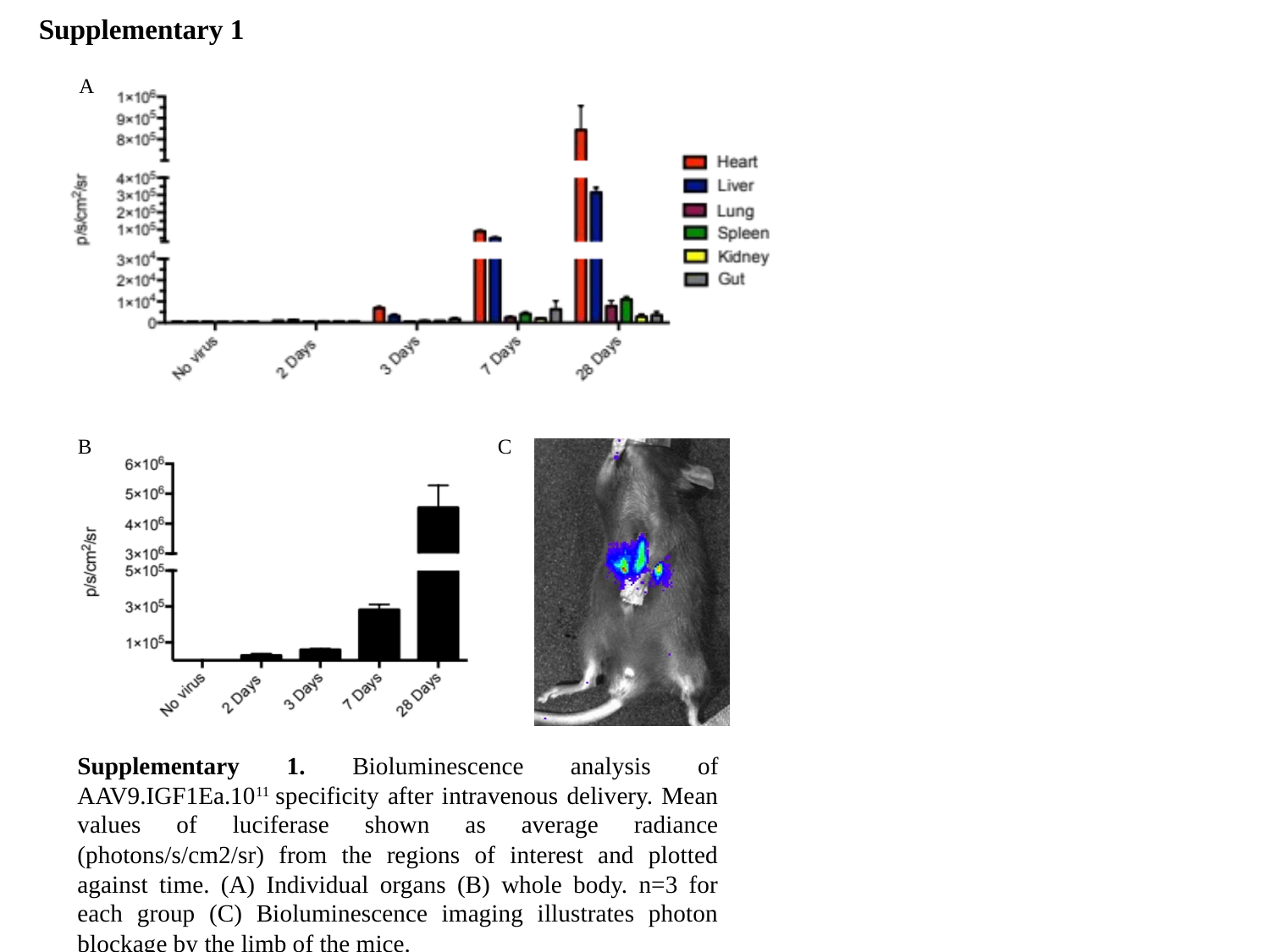

Supplementary 1
A
B
C
Supplementary 1. Bioluminescence analysis of AAV9.IGF1Ea.1011 specificity after intravenous delivery. Mean values of luciferase shown as average radiance (photons/s/cm2/sr) from the regions of interest and plotted against time. (A) Individual organs (B) whole body. n=3 for each group (C) Bioluminescence imaging illustrates photon blockage by the limb of the mice.

## Slide 2
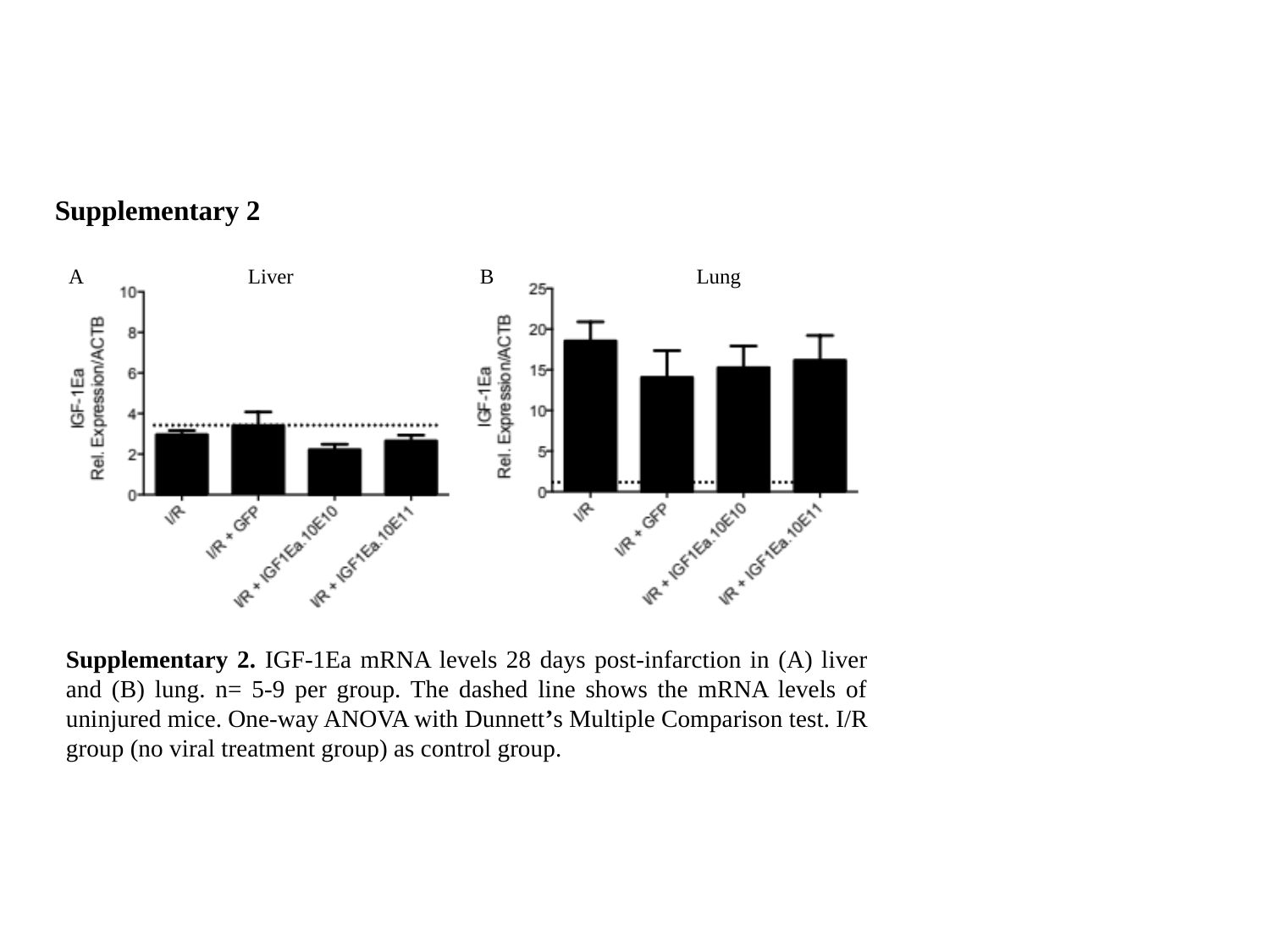

Supplementary 2
A
Liver
B
Lung
Supplementary 2. IGF-1Ea mRNA levels 28 days post-infarction in (A) liver and (B) lung. n= 5-9 per group. The dashed line shows the mRNA levels of uninjured mice. One-way ANOVA with Dunnett’s Multiple Comparison test. I/R group (no viral treatment group) as control group.

## Slide 3
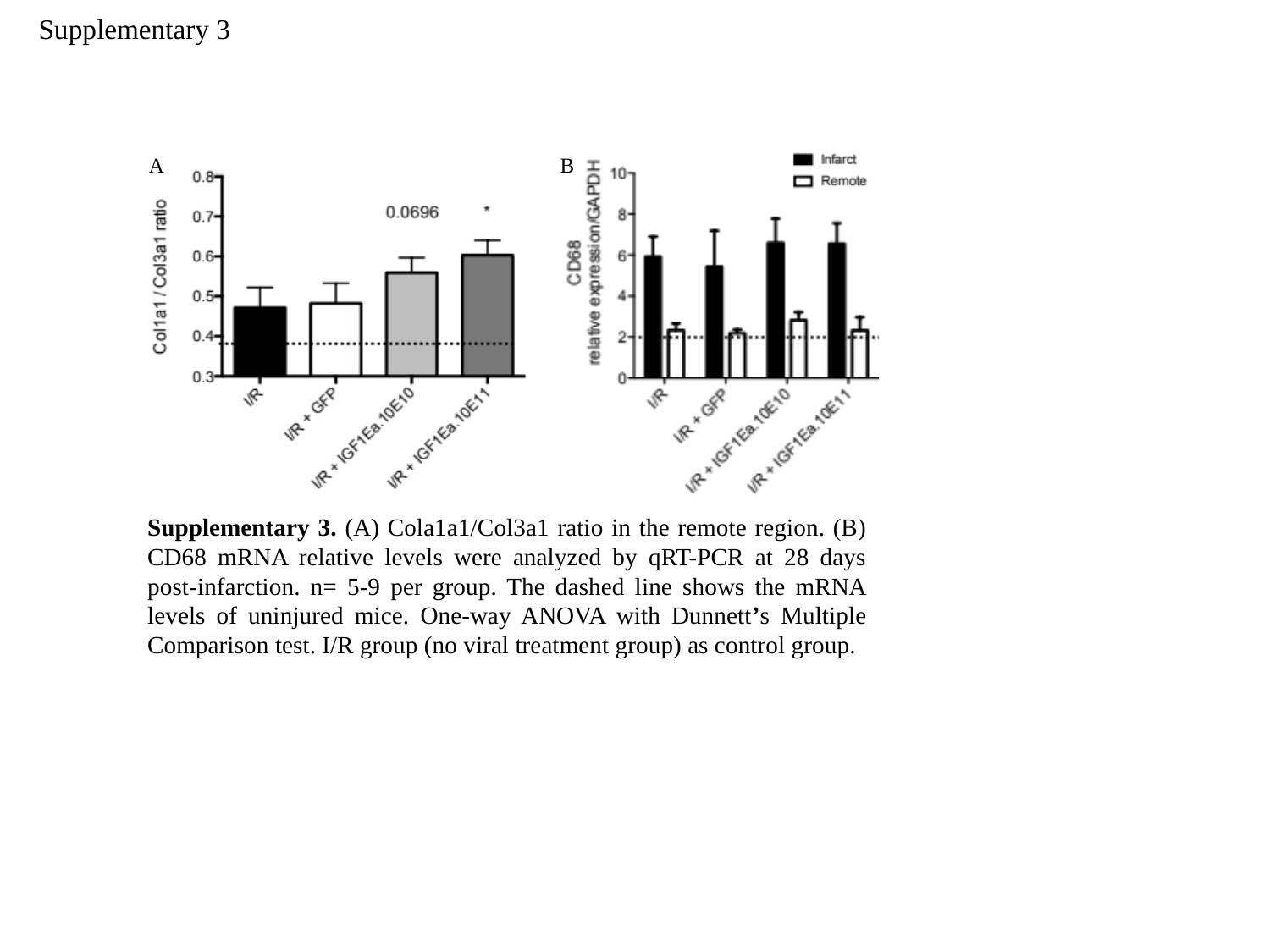

Supplementary 3
A
B
Supplementary 3. (A) Cola1a1/Col3a1 ratio in the remote region. (B) CD68 mRNA relative levels were analyzed by qRT-PCR at 28 days post-infarction. n= 5-9 per group. The dashed line shows the mRNA levels of uninjured mice. One-way ANOVA with Dunnett’s Multiple Comparison test. I/R group (no viral treatment group) as control group.

## Slide 4
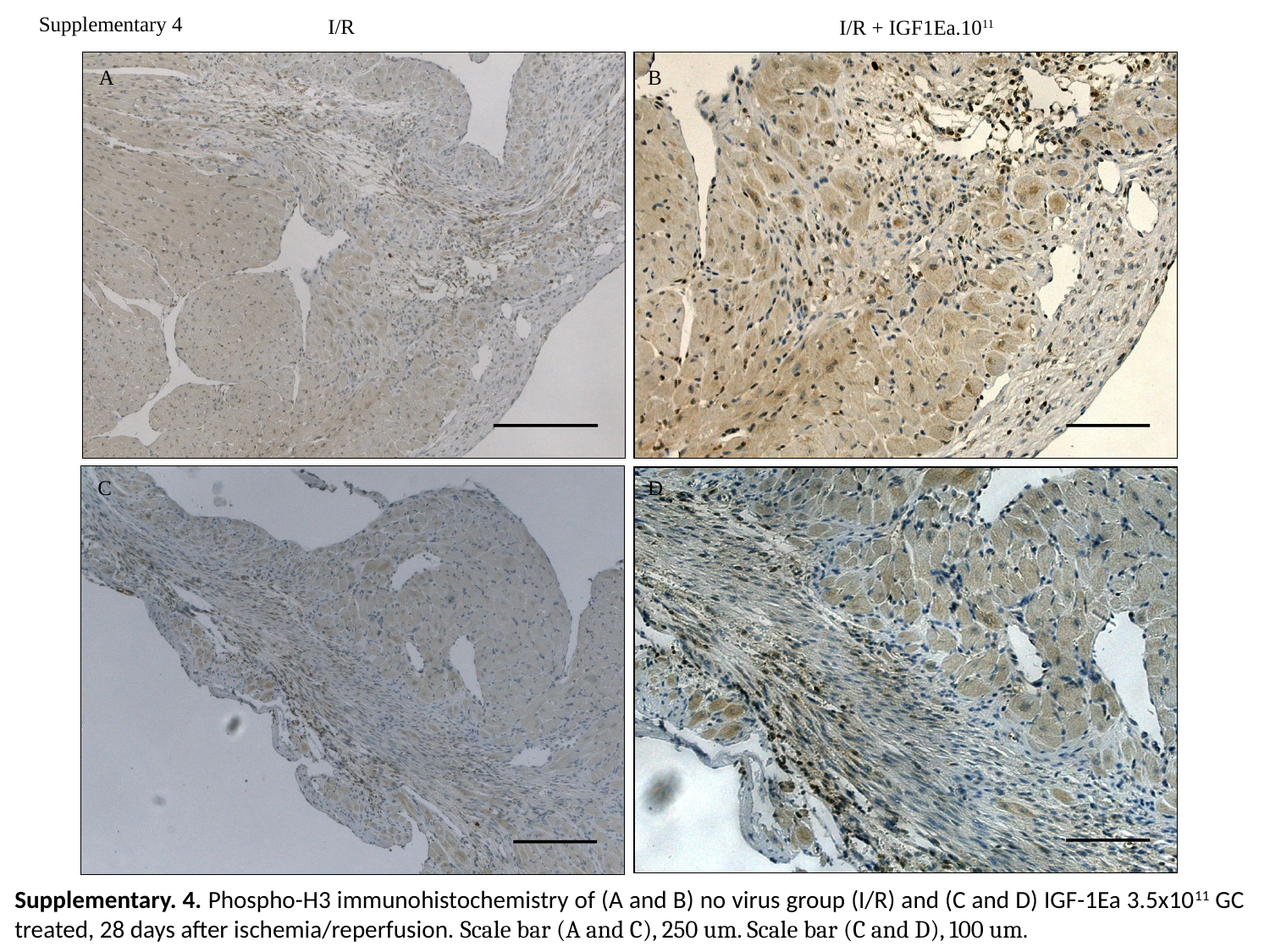

Supplementary 4
I/R
I/R + IGF1Ea.1011
A
A
B
C
D
Supplementary. 4. Phospho-H3 immunohistochemistry of (A and B) no virus group (I/R) and (C and D) IGF-1Ea 3.5x10^11^ GC treated, 28 days after ischemia/reperfusion. Scale bar (A and C), 250 um. Scale bar (C and D), 100 um.
